# Increased oxidative stress and coenzyme Q10 deficiency in centenarians

**DOI:** 10.3164/jcbn.17-124

**Published:** 2018-03-17

**Authors:** Midori Nagase, Yorihiro Yamamoto, Nozomi Matsumoto, Yasumichi Arai, Nobuyoshi Hirose

**Affiliations:** 1 School of Bioscience and Biotechnology, Tokyo University of Technology, 1404-1 Katakura-cho, Hachioji, Tokyo 192-0982, Japan; 2 Center for Supercentenarian Medical Research, Keio University School of Medicine, 35 Shinanomachi, Shinjuku-ku, Tokyo 160-8582, Japan

**Keywords:** coenzyme Q10, cholesterol metabolism, free fatty acids and their composition, centenarians, prosaposin

## Abstract

Aging populations are expanding worldwide, and the increasing requirement for nursing care has become a serious problem. Furthermore, successful aging is one of the highest priorities for individuals and societies. Centenarians are an informative cohort to study and inflammation has been found to be a key factor in predicting cognition and physical capabilities. Inflammation scores have been determined based on the levels of cytokines and C-reactive protein, however, serum antioxidants and lipid profiles have not been carefully examined. We found that the redox balance of coenzyme Q10 significantly shifted to the oxidized form and levels of strong antioxidants, such as ascorbic acid and unconjugated bilirubin, decreased significantly compared to 76-year-old controls, indicating an increased oxidative stress in centenarians. Levels of uric acid, an endogenous peroxynitrite scavenger, remained unchanged, suggesting that centenarians were experiencing moderate, chronic inflammatory conditions. Centenarians exhibited a hypocholesterolemic condition, while an increase in the ratio of free cholesterol to cholesterol esters suggests some impairment of liver function. Serum free fatty acids and monoenoic acid composition, markers of tissue oxidative damage, were significantly decreased in centenarians, indicating an impairment in the tissue repair system. Despite an elevation of the coenzyme Q10 binding protein Psap, serum total coenzyme Q10 levels decreased in centenarians. This suggests a serious deficiency of coenzyme Q10 in tissues, since tissue levels of coenzyme Q10 significantly decrease with age. Therefore, coenzyme Q10 supplementation could be beneficial for centenarians.

## Introduction

Aging populations are expanding worldwide, and the increasing requirement for nursing care has become a serious problem. Successful aging without cognition loss and physical deficiencies is one of the highest priorities for individuals and societies. Arai *et al.*^(1)^ found that only 20% of centenarians enjoyed physical and cognitive independence at the age of 100 years, although most remained independent in daily living into their 90s. Those who maintained physical independence at 100 years of age were highly likely to become semi-supercentenarians (over 105 years) or supercentenarians (beyond 110 years).^(1)^

To identify key factors in successful aging, Arai *et al.*^(2)^ focused on the characteristics of centenarians, semi-supercentenarians, and supercentenarians. They found that inflammation predicted cognition and physical capabilities in (semi-) supercentenarians better than chronologic age or gender. Interestingly, the inflammation score was lower in centenarian offspring compared to age-matched controls.^(2)^ They concluded that inflammation is an important malleable driver of aging up to extreme old age in humans.^(2)^ Other reviews also emphasize that the suppression of chronic inflammation is an important driver of successful aging at extreme old age.^(1,3,4)^

In the above study, an inflammation score was estimated using cytomegalovirus immunoglobulin G antibody titers and plasma levels of interleukin- 6, tumor necrosis factor-α, and C-reactive protein.^(2)^ Acute inflammation, such as sepsis, is characterized by the formation of reactive oxygen and nitrogen species such as superoxide and nitric oxide.^(5–7)^ Therefore, peroxynitrite is also an important reactive molecule since it is produced from the combination of superoxide and nitric oxide.^(5–7)^ In fact, we observed a decline in plasma antioxidants, namely vitamin E (VE), ubiquinol-10 (CoQ10H_2_), vitamin C (VC), and uric acid (UA), in patients with sepsis.^(8)^ However, no comprehensive study has been reported for centenarians. In this study, we compared serum levels of antioxidants in centenarians and 76-year-old controls. We found a significant decrease in VC and unconjugated bilirubin (BR) and a significant increase in the percentage (%CoQ10) of the oxidized form of coenzyme Q10 (CoQ10) to total coenzyme Q10 (TQ10) in centenarians, suggesting an increase of oxidative stress.

The plasma levels of high density lipoprotein (HDL) were reported to be decreased in centenarians.^(2)^ We, therefore, measured serum free cholesterol (FC) and cholesterol esters (CE) because their ratio (FC/CE) is determined by the activity of lecithin-cholesterol acyltransferase (LCAT) secreted with HDL from liver.^(9,10)^ We confirmed a significant decrease in CE and total cholesterol (TC) and a significant increase in FC/CE ratio in centenarians.

We also measured serum free fatty acids (FFA) and the content of oxidatively vulnerable polyunsaturated fatty acids in total FFA (%PUFA) as markers of tissue oxidative damage.^(11)^ It is common that stearoyl-CoA desaturase is activated to compensate for the loss of PUFA; therefore, the percentages of palmitoleic acid and oleic acid in total FFA (%16:1 and %18:1, respectively) are also appropriate markers of tissue oxidative damage.^(11)^

It is well known that human tissue levels of TQ10 decrease with age after the age of 20.^(12)^ For example, decreases in TQ10 of >30% and 50% in human heart were observed at ages 40 and 80, respectively.^(12)^ However, such a decline of TQ10 was not observed in human plasma within the range of 20 to 60.^(13)^ In this study, we found a significant decrease in serum TQ10 levels in centenarians as compared with 76-year-old controls. On the other hand, a significant increase of the coenzyme Q10 binding and transfer protein prosaposin (Psap)^(14–16)^ was observed. The possible role of Psap will be discussed.

## Materials and Methods

### Study design

This study comprised 99 Japanese centenarians (25 males aged 100.8 ± 1.3 years and 74 females aged 100.9 ± 1.5 years) and 62 Japanese controls (25 males aged 74.6 ± 8.5 years and 37 females aged 76.2 ± 8.0 years). The above 4 groups are abbreviated as 101M, 101F, 75M and 76F, respectively. Written informed consent to participate was obtained either from the participants or a proxy when individuals lacked the capacity to consent. The protocol of this study was approved by the Ethical Committee of the Keio University School of Medicine. Non-fasting venous blood was sampled. Serum was collected and stored at −80°C until analysis. We also compared with previously reported 60-year-old controls^(17)^ (consisting of 38 males and 17 females, 60.1 ± 9.3 years).

### Analytical procedures

Serum levels of VE, CoQ10H_2_, CoQ10, FC and CE were determined as previously described with some modifications.^(18)^ In brief, serum was extracted with 19 volumes of 2-propanol and the extract was analyzed by HPLC using two analytical columns (Supelcosil ABZ+, 3 µm, 3.3 cm × 4.6 mm i.d. and Ascentis LC-8, 5 µm, 25 cm × 4.6 mm i.d.; Supelco Japan, Tokyo, Japan) connected in tandem, a reduction column (RC-10-1; Irica, Kyoto, Japan) and an amperometric electrochemical detector (Model Σ985; Irica) with an oxidation potential of +600 mV (vs Ag/AgCl) on a glass carbon electrode. The mobile phase consisted of 50 mM sodium perchlorate in methanol/2-propanol (78/22, v/v), delivered at a flow rate of 0.8 ml/min. The analytical columns were cooled to 25°C.

Serum levels of VC, UA and BR were determined by HPLC on a bonded-phase aminopropylsilyl column (Supelcosil LC-NH_2_, 5 µm, 25 cm × 4.6 mm i.d.; Supelco Japan) with UV/VIS detection (265 nm for 0–15 min and 460 nm for 15–22 min), as described previously.^(19)^

Serum FFA were derivatized with monodansylcadaverine for analysis by HPLC.^(20)^ Briefly, serum samples (50 µl) were mixed with 200 µl of methanol and then centrifuged at 13,000 × *g* for 5 min. Aliquots (50 µl) of supernatants were mixed with 20 µl of methanol containing 25 µM tridecanoic acid (internal standard) and dried under a stream of nitrogen gas, and the residue was admixed with diethyl phosphorocyanidate (1 µl) and *N*,*N*-dimethylformamide (50 µl) containing monodansylcadaverine (2 mg/ml) and kept at room temperature in the dark for 20 min. A 5-µl sample was injected onto an octadecylsilyl column (3 µm, 3.3 cm × 4.6 mm i.d.; Supelco Japan) and a pKb-100 column (5 µm, 25 cm × 4.6 mm i.d.; Supelco Japan) connected in tandem. The FFA components were measured by fluorescence detection (Model 821-FP; Japan Spectroscopic, Tokyo, Japan) with excitation at 320 nm and emission at 520 nm. The mobile phase consisted of acetonitrile/methanol/water (17.5/65.0/17.5, v/v/v) delivered at a flow rate of 1.5 ml/min. The analytical columns were heated to 40°C.

Serum levels of Psap were measured by a sandwich ELISA using monoclonal and polyclonal antibodies against human saposin B.^(14)^ Plasma was diluted 100 times with a phosphate-buffer saline containing 0.1% Triton X-100, 1 g/L NaN_3_, 10 g/L BSA, and 1 mM EDTA. Purified saposin B was used as a standard.^(14)^

### Statistical analysis

Data are presented as mean values with standard deviations. Statistical analysis was performed using one-way ANOVA followed by the Scheffe’s multiple comparisons test. *P*<0.05 was considered statistically significant.

## Results and Discussion

### Serum antioxidants and oxidative stress

**Fig. 1:**
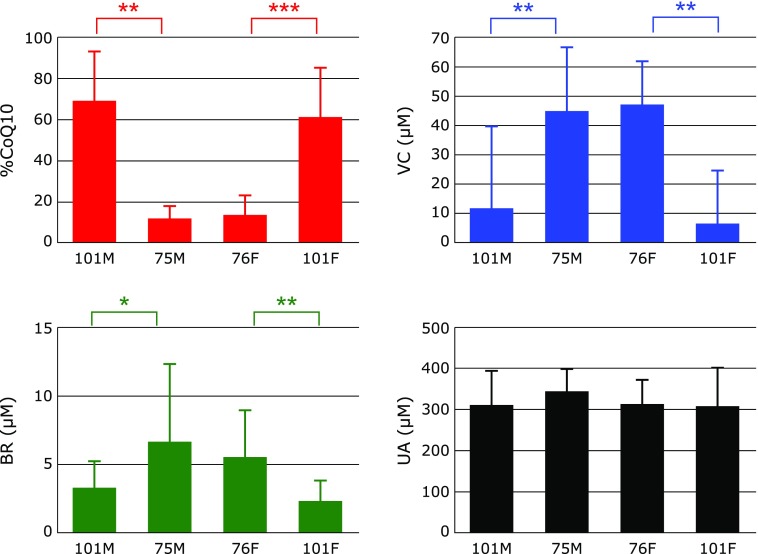
Comparison of the percentage of oxidized coenzyme Q10 to total coenzyme Q10 (%CoQ10) in serum, and serum levels of ascorbic acid (VC), unconjugated bilirubin (BR), and uric acid (UA) among male centenarians (101M), male 75-year-old controls (75M), female 76-year-old controls (76M), and female centenarians (101F). Data are presented as mean + SD. *p<0.01 and **p<0.001, significant differences as determined by Scheffe’s multiple comparison test.

Figure 1 shows serum %CoQ10 and serum levels of VC, BR, and UA among male and female centenarians (101M and 101F, respectively) and 75-year-old male and 76-year-old female controls (75M and 76F, respectively). There were no significant differences between the male and female groups in each age category. However, a significant increase in %CoQ10 was observed in centenarians compared with 76-year-old controls, indicating that the redox balance of coenzyme Q10 shifted to the oxidized form. This confirms an increase in oxidative stress in centenarians and agrees with a significant decrease in serum antioxidants such VC^(11)^ and BR^(21)^ in centenarians. These results are also consistent with the observation that chronic inflammation is present in centenarians.^(2)^

Under acute inflammatory conditions like sepsis, the substantial formation of superoxide and nitric oxide, and their product peroxynitrite, is expected. In fact plasma UA levels declined significantly in patients with sepsis during a stay at an intensive care unit^(8)^ because UA is a good inhibitor of peroxynitrite.^(22–24)^ However, serum UA levels remained constant in centenarians and 76-year-old controls, suggesting that inflammation in centenarians is moderate and chronic.

**Fig. 2:**
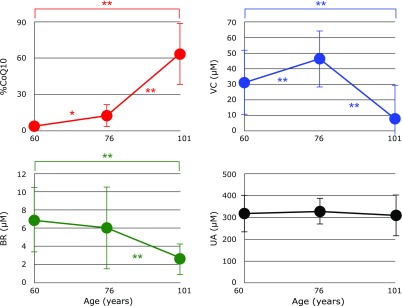
Changes in the percentage of oxidized coenzyme Q10 to total coenzyme Q10 (%CoQ10) in serum, and serum levels of ascorbic acid (VC), unconjugated bilirubin (BR), and uric acid (UA) with age. Data are presented as mean ± SD. *p<0.05 and **p<0.001, significant differences as determined by Scheffe’s multiple comparison test.

Since there were no significant differences in %CoQ10, VC, BR, and UA between the male and female groups of centenarians and 76-year-old controls, the data were combined into centenarians and 76-year-old controls (abbreviated as 101 and 76) and compared with 60-year-old controls^(17)^ as shown in Fig. 2. %CoQ10 consistently increased with age, while levels of VC and BR in centenarians were significantly lower than 60- or 76-year-old controls. These data confirm that centenarians are under oxidative stress. However, levels of UA remained unchanged, suggesting that the formation of peroxynitrite is not very significant in centenarians and they are under moderate, chronic inflammatory conditions.

### Serum levels of cholesterols

**Fig. 3:**
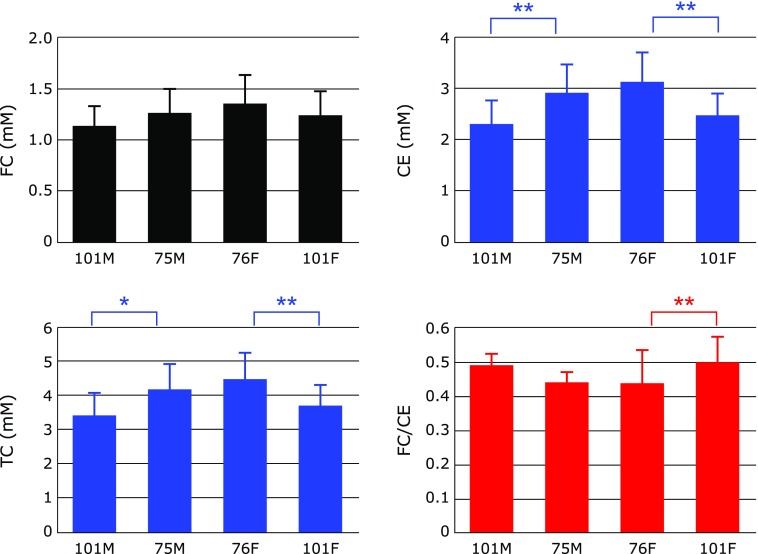
Comparison of serum free cholesterol (FC), cholesterol esters (CE), total cholesterol (TC), and the FC/CE ratio among male centenarians (101M), male 75-year-old controls (75M), female 76-year-old controls (76M), and female centenarians (101F). Data are presented as mean + SD. *p<0.01 and **p<0.001, significant differences as determined by Scheffe’s multiple comparison test.

**Fig. 4:**
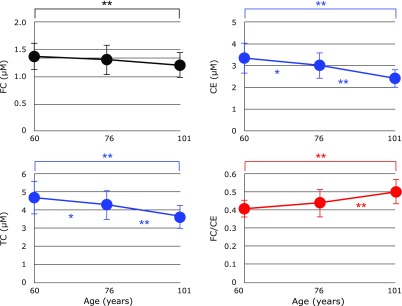
Changes in serum free cholesterol (FC), cholesterol esters (CE), total cholesterol (TC), and the FC/CE ratio with age. Data are presented as mean ± SD. *p<0.01 and **p<0.001, significant differences as determined by Scheffe’s multiple comparison test.

Figure 3 shows serum levels of FC, CE and TC, as well as the FC/CE ratio. A slight decrease in FC was observed in centenarians, however the difference was not significant. A significant decrease in CE and TC was observed, resulting in an increase in the FC/CE ratio. There were no significant differences in the levels of FC, CE and TC, and the FC/CE ratio between male and female groups in each age category. Thus, we plotted the pooled data against age (Fig. 4). FC, CE and TC were all observed to decrease with age. Since the decline of CE was more profound than FC, the FC/CE ratio increased with age. The FC/CE ratio is determined by the activity of LCAT secreted with HDL from liver.^(9,10)^ Therefore, these data indicate a degree of impairment in the secretion of LCAT with HDL and liver function. This is consistent with the previous observation that serum levels of HDL are low in centenarians.^(2)^

### Serum FFA composition and tissue oxidative damage

**Fig. 5:**
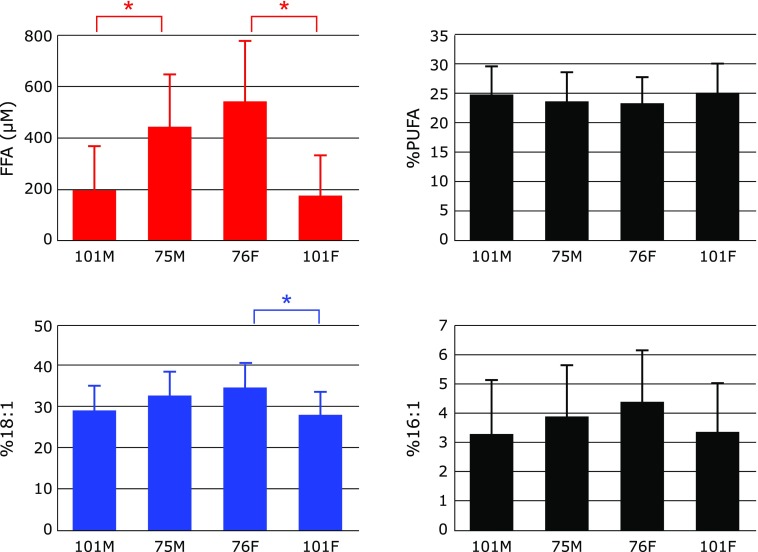
Comparison of serum free fatty acids (FFA), the percentage of polyunsaturated fatty acids in total FFA (%PUFA), the percentage of palmitoleic acid in total FFA (%16:1), and the percentage of oleic acid in total FFA (%18:1) among male centenarians (101M), male 75-year-old controls (75M), female 76-year-old controls (76M), and female centenarians (101F). Data are presented as mean + SD. *p<0.001, significant differences as determined by Scheffe’s multiple comparison test.

Figure 5 shows serum levels of FFA, %PUFA, %18:1 and %16:1. A significant decrease in FFA was observed in centenarians. Under oxidative stress, plasma levels of FFA have been observed to increase in many cases, such as in newborn babies^(25)^ and patients with hepatitis,^(11)^ cirrhosis,^(11)^ hepatoma, ^(11)^ juvenile fibromyalgia,^(26)^ and post-cardiac arrest syndrome.^(27)^ Elevated plasma FFA was observed in the rat middle cerebral artery occlusion model of stroke.^(28)^ It is of interest that repeated administration of the antioxidant edaravone significantly improved the neurological symptoms and impairment of motor function induced by a middle cerebral artery occlusion, and reduced the levels of FFA to those of a sham operation.^(28)^ Formation of FFA under oxidative stress is assumed to be a result of phospholipase activity,^(29–32)^ therefore, a significant decrease in serum FFA in centenarians must be ascribed to impairment of the repair system that counteracts increases in oxidative stress.

Under conditions of elevated oxidative stress, oxidatively vulnerable PUFA is selectively damaged which results in decreased membrane fluidity.^(11)^ To compensate for the loss of PUFA, stearoyl-CoA desaturase is activated and converts stearic and palmitic acids to 18:1 and 16:1, respectively.^(33)^ Accordingly, a decrease in %PUFA and an increase in %18:1 and %16:1 have been observed under oxidative stress.^(11,25–28)^

**Fig. 6:**
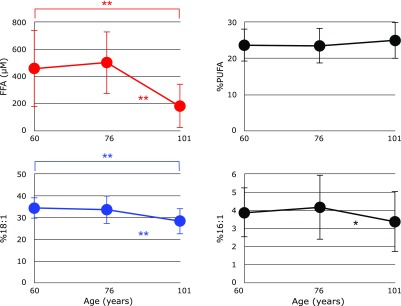
Changes in serum free fatty acids (FFA), the percentage of polyunsaturated fatty acids in total FFA (%PUFA), the percentage of palmitoleic acid in total FFA (%16:1), and the percentage of oleic acid in total FFA (%18:1) with age. Data are presented as mean ± SD. *p<0.05 and **p<0.001, significant differences as determined by Scheffe’s multiple comparison test.

Since there were no significant differences in FFA levels, %PUFA, %18:1, and %16:1 between the male and female groups in each age category, we plotted the pooled data against age (Fig. 6). No significant changes in %PUFA were observed in centenarians. In contrast, significant decreases in %18:1 and %16:1 were observed in centenarians, indicating impairment in the oxidative repair system. However, this hypothesis should be investigated further.

### Serum TQ10 and Psap

**Fig. 7:**
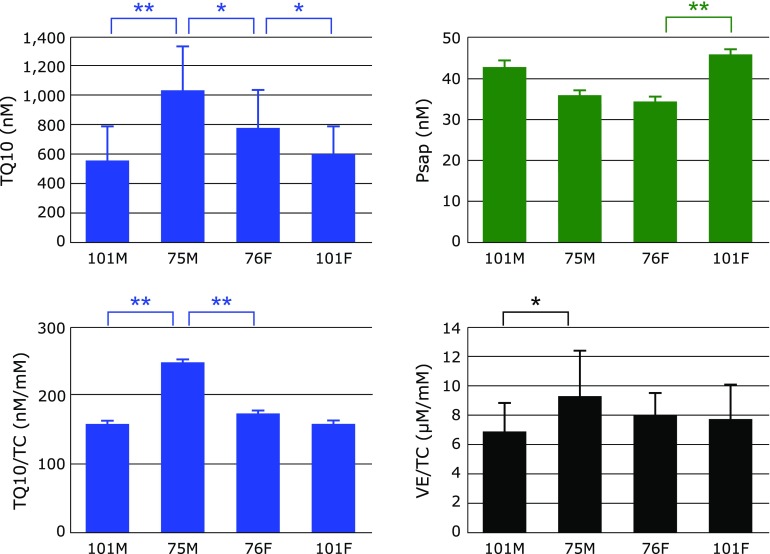
Comparison of serum total coenzyme Q10 (TQ10), the ratio of vitamin E to total cholesterol (VE/TC), the ratio of TQ10/TC, and serum prosaposin (Psap) among male centenarians (101M), male 75-year-old controls (75M), female 76-year-old controls (76M), and female centenarians (101F). Data are presented as mean + SD. *p<0.01 and **p<0.001, significant differences as determined by Scheffe’s multiple comparison test.

Figure 7 shows serum levels of TQ10 and Psap, as well as the ratio of VE/TC and TQ10/TC in centenarians and 76-year-old controls. A significant decrease in TQ10 was observed in centenarians compared with 76-year-old controls, suggesting a coenzyme Q10 deficiency in centenarians. This is also the case in male centenarians even if TQ10 was normalized to TC, however, female centenarians were not significantly different. A similar trend was observed in VE/TC values.

**Fig. 8:**
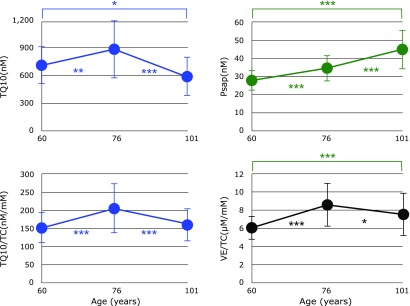
Changes in serum total coenzyme Q10 (TQ10), the ratio of vitamin E to total cholesterol (VE/TC), the ratio of TQ10/TC, and serum prosaposin (Psap) with age. Data are presented as mean ± SD. *p<0.05, **p<0.01 and ***p<0.001, significant differences as determined by Scheffe’s multiple comparison test.

On the other hand, a significant increase in coenzyme Q10 binding and transfer protein (Psap)^(14–16)^ was observed in female centenarians compared with 76-year-old female controls, and male centenarians showed a similar tendency. Figure 8 shows the combined male and female data of 60- and 76-year-old controls, and centenarians. Psap levels increased progressively and significantly with age while TQ10 levels and the TQ10/TC ratio reached a maximum at 76 years and subsequently decreased. It is well known that tissue TQ10 levels decrease with age; for example >30% and 50% decreases in TQ10 were observed at the ages of 40 and 80, respectively, in human heart.^(12)^ Furthermore, the rate of coenzyme Q biosynthesis in rat heart is much less than that in rat kidney.^(34)^ This should be also the case in human. These observations suggest that the human heart in octogenarians has a serious requirement for exogenous TQ10. Therefore, coenzyme Q10 should be transferred from its pool (most likely to be kidney) to heart using Psap. This is a likely explanation for the observed increase in serum Psap levels in 76-year-olds, and the consequent increase in serum TQ10 levels, compared with 60-year-old controls.

In centenarians, it is reasonable for Psap levels to increase in order to compensate for the loss of coenzyme Q10. Despite further elevation of Psap in centenarians, their TQ10 levels were decreased, indicating their tissue TQ10 levels were likely to be critically low.

Coenzyme Q10 is essential for ATP production in the mitochondria and is an important antioxidant in every cellular membrane and lipoprotein.^(35)^ Its importance is also suggested by the observation that serum coenzyme Q10 levels were inversely associated with the risk of disabling dementia.^(36)^ Furthermore, a mutation in the coenzyme Q10 synthesis enzyme was identified in patients with familial multiple system atrophy (MSA).^(37)^ Plasma levels of coenzyme Q10 in patients with MSA were significantly lower than controls.^(38,39)^ Now the phase 2 clinical study of coenzyme Q10 supplementation to patients with MSA is on going. Therefore, coenzyme Q10 supplementation would be beneficial for centenarians, although this hypothesis requires further serious investigation.

## Conclusion

Oxidative stress in centenarians was demonstrated as an increase in serum %CoQ10 and a decrease in VC compared with 76-year-old controls. Centenarians are suggested to exist in a moderate, chronic inflammatory condition because serum levels of UA were similar to those in 76-year-old controls. Centenarians exhibit a hypocholesterolemic condition and the observed increase in the FC/CE ratio suggests some impairment of liver function. A significant decrease in serum FFA, %18:1 and %16:1 also indicates impairment of the tissue repair system in centenarians. Despite an elevation of the coenzyme Q10 binding protein Psap, serum TQ10 levels decreased in centenarians, suggesting a serious TQ10 deficiency in tissues. Therefore, coenzyme Q10 supplementation is likely to be beneficial for centenarians.

## Abbreviations

%16:1: percentage of palmitoleic acid in total FFA
%18:1: percentage of oleic acid in total FFA
%CoQ10: percentage of oxidized form of coenzyme Q10 in TQ10
%PUFA: percentage of polyunsaturated fatty acids in total FFA
BR: unconjugated bilirubin
CE: cholesterol esters
CoQ10: oxidized form of coenzyme Q10
CoQ10H_2_: ubiquinol-10, reduced form of coenzyme Q10
FC: free cholesterol
FFA: free fatty acids
HDL: high density lipoprotein
LCAT: lecithin-cholesterol acyltransferase
MSA: multiple system atrophy
Psap: prosaposin
TC: total cholesterol
TQ10: total coenzyme Q10
UA: uric acid
VC: vitamin C
VE: vitamin E
